# Paediatric Autoimmune Neuropsychiatric Disorder Associated with Group A Beta-Haemolytic Streptococcal Infection: An Indication for Tonsillectomy? A Review of the Literature

**DOI:** 10.1155/2018/2681304

**Published:** 2018-02-21

**Authors:** Amarkumar Dhirajlal Rajgor, Navid Akhtar Hakim, Sanah Ali, Adnan Darr

**Affiliations:** ^1^University Hospital North Midlands, Royal Stoke University Hospital, Stoke-on-Trent, UK; ^2^Russells Hall Hospital, Dudley, UK

## Abstract

*Background. *Paediatric Autoimmune Neuropsychiatric Disorder Associated with Streptococcal Infection (PANDAS) is the acute onset of neuropsychiatric symptoms following group A beta-haemolytic streptococcal infection. The aetiology remains elusive. However, with group A streptococcus being the most common bacterial cause of tonsillitis, surgical intervention in the form of tonsillectomy has often been considered as a potential therapy.* Methods.* A MEDLINE® search was undertaken using keywords “PANDAS” or “paediatric autoimmune neuropsychiatric disorders associated with streptococcus” combined with “tonsillectomy”.* Results*. Six case reports and 3 case series met the inclusion criteria. Demesh et al. (case series) reported a dramatic reduction in neuropsychiatric symptom severity in the patient cohort undergoing tonsillectomy. Two case series suggest that there is no association between tonsillectomy and resolution of PANDAS.* Conclusion*. Due to the lack of uniform data and sporadic reports, tonsillectomy should be carefully adopted for the treatment of this disorder. In particular, tonsillectomies/adenoidectomies to alleviate neuropsychiatric symptoms should be avoided until more definitive evidence is at our disposal. This review highlights the importance of a potential collaborative prospective study.

## 1. Introduction

Group A beta-haemolytic streptococcal (GABHS) tonsillitis, more frequently known as streptococcal pharyngitis, is highly prevalent in children especially in those who are between the ages of 5 and 15 years [1]. It is the commonest bacterial cause of tonsillitis in both children and adults, accounting for approximately 30% of all cases [2], with at least a single infection before the age of 5 [3]. Furthermore, several studies have suggested that as many as half of children presenting with pharyngitis could be GABHS carriers, making virtuously impossible to distinguish these children from those with the actual disease just based on signs and symptoms alone [4–9].

Paediatric Autoimmune Neuropsychiatric Disorder Associated with Streptococcal Infection (PANDAS) is the acute onset of neuropsychiatric symptoms following GABHS infection. PANDAS was initially described by Swedo et al. in 1998 following a study of 50 patients who had developed obsessive-compulsive disorder (OCD) and/or tics following a poststreptococcal autoimmune process [10, 11]. Despite much research, the aetiology of PANDAS is still unclear, with several hypotheses suggesting a variety of genetic and environmental factors as well as autoimmune mechanisms [12]. The incidence and prevalence of PANDAS remain uncertain [13] but some studies suggest that it could play a role in more than 10% of childhood-onset OCD [14] and tics [15].

Group A streptococcal infection pathogenesis is described in three stages [16, 17]. Initially there is adherence to the pharyngeal epithelium where pili-like cell surface structures play a key role in pharyngeal epithelial cell adherence and biofilm formation [18, 19]. Next, nutrients based on carbon sources are attained for proliferation, which triggers the clinical features found in pharyngitis [20, 21]. This is followed by group A streptococcus being able to avoid the host's innate immune response by secreting proteins that inhibits phagocytosis and polymorphonuclear- (PMN-) mediated killing [22, 23]. Evidence suggests that PANDAS is most probably an autoimmune phenomenon involving pathogenic autoantibodies [24]. However, to date, the biological value of detected antineuronal antibodies remains to be determined [10, 25]. The commonest hypothesis is that of molecular mimicry where it is thought the autoantibodies that target brain structures are abnormally produced [1, 2]. Other mechanisms such as epitope spreading and bystander activation also seem to play a role in the production of defective autoantibody production [4, 5, 26]. Human basal ganglia antigens have been demonstrated in patients with encephalitis and movement disorders in 2004 [27] but the clinical relevance was not widely accepted due to methods used (western blotting of soluble brain extracts) [28]. Subsequently an intracellular enzyme was identified as a target antigen of these antibasal ganglia autoantibodies in 2006 [29]. In 2012, it was reported that both dopamine-1 receptor (D1R) and dopamine-2 receptor (D2R) antibodies were detected in patients with Sydenham's chorea and PANDAS [30] but methods used (ELISA and western blotting) were again not optimal for cell surface antigen detection [31]. These antibodies bind to the neuronal surface and cause elevated calcium/calmodulin-dependent protein kinase II signalling in neuronal cell lines [26]. Dale et al. later implemented a flow-cytometry based approach to identify autoantibodies against neuronal surface antigenic targets as this was a more specific methodology, but none were detected in the PANDAS group [32].

Tonsillitis remains a clinical diagnosis. To distinguish between a viral and a bacterial origin, laboratory testing such as throat swab culture, rapid antigen detection testing (RADT), or antistreptolysin titres (ASOT) is necessary. If performed correctly, throat swab cultures grown on a blood agar plate have a sensitivity of 90–95% in detecting GABHS [33]. This remains the standard test used despite the disadvantage of time constraints for results analysis [34]. RADT is faster but more expensive, with a specificity of 95%, but a sensitivity of 80–90% [35]. This means there is a chance of false negative results with RADT, which could result in misdiagnosis and group A streptococcal spread and even rarely lead to an increased risk of suppurative and nonsuppurative complications [36]. Antibodies against streptokinase (ASK), streptolysin O (ASO), and deoxyribonuclease B (anti-DNAseB) are not reliable markers for acute GABHS infection, used either alone or in combination with one another [37]. According to the National Institute of Mental Health Clinical Diagnostic Criteria for PANDAS, the diagnosis of PANDAS is based on five criteria (presence of OCD and/or a tic disorder, prepubertal onset, abrupt onset and relapsing-remitting course, association with GABHS infection, and association with neurological abnormalities) [10, 38–41].

## 2. The Role of Surgical Intervention in PANDAS

Surgical intervention by means of tonsillectomy or adenoidectomy has long been considered as an alternative to pharmacological therapy in the management of PANDAS [42]. Literature advocating such management appears to have increased despite the ongoing need for robust evidence. Despite the fact that many PANDAS patients meet the globally acknowledged criteria for tonsillectomy (≥7 severe episodes of tonsillitis within the preceding year, ≥5 within the preceding 2 years or, ≥3 within the preceding 3-year period) and therefore PANDAS generally represents a comorbidity in these cases, a great deal of disagreement as to the efficacy of this treatment option in providing remission or symptoms improvement still exists due to insufficient evidence [42]. The role of tonsillectomy in PANDAS has predominantly been evoked by case reports citing a reduction in frequency of tics as well as OCD after surgery [43, 44].

Three case series and six case reports met the inclusion criteria. The results are summarized in Tables 1 and 2, respectively.

## 3. Method

A review of the literature was performed to evaluate the published evidence regarding surgical management (tonsillectomy) of PANDAS. A structured search of the MEDLINE online database (no interval/period was stipulated) was undertaken. Keywords “PANDAS” or “paediatric autoimmune neuropsychiatric disorders associated with streptococcus” were combined with “tonsillectomy”. The criteria for inclusion of citations were English language case reports and case series with confirmed and definitive cases of PANDAS according to the Institute of Mental Health Clinical Diagnostic Criteria for PANDAS. References of all the subsequent articles including case series were also evaluated for further case reports relevant to the subject. Information including clinical presentation, ASOT titre, medication attempted, follow-up time, and overall outcome was recorded from the details provided in each case report.

## 4. Discussion

With regard to case series, Murphy et al. [45] played a crucial role in refuting the notion that tonsillectomy may play a protective role in the development of the disease. Using the DSM-IV-R criteria for OCD/tic disorders, 112 patients were included in a prospective study to ascertain if a correlation existed between ASO titres in surgical patients (*n* = 36), as well as symptomology. Of the 112 patients, 43 had confirmed PANDAS. More than 50% of cases developed neuropsychiatric symptoms within 2.4–2.9 years after tonsillectomy. Also, the resultant incidence of PANDAS was almost twice that of the nonsurgical cohort. Interestingly, 46.5% of the PANDAS population underwent surgery compared to only 23.2% of the non-PANDAS population. Although PANDAS is not an indication for tonsillectomy, this may demonstrate the potential influence that neuropsychiatric symptoms may have on a clinicians' decision to operate. This study has numerous limitations. The study design only included children with current neuropsychiatric symptoms that were retrospectively stratified based on whether a tonsillectomy was performed. Therefore, in case of postoperative remission, a child would not have entered the study. In addition, the ASO titres were assessed at the beginning of the study rather than verifying a temporal relationship with OCD/tic occurrence. ASO titres have been shown to vary with age and are more representative of an infection if serial measures are performed [46]; thus it would have been more valuable to undertake serial titres and adjust them for age.

Another prospective nonrandomized case series was reported by Pavone et al. (2014) [47]. The study included 120 patients with PANDAS who either had undergone tonsillectomy or adenotonsillectomy (*n* = 56) or had been managed conservatively (*n* = 64). Patients were followed up for 24 months. Clinical progression, antibody production, and neuropsychiatric symptomatology did not differ in the two groups. Importantly, all assessments of symptoms were made using the validated Children's Yale-Brown Obsessive Compulsive Scale (CY-BOCS) and Yale Global Tic Severity Scale (YDTSS) by appropriately trained clinicians. However, Pavone et al. did not account for the several factors that can influence titres such as age. Both the antibody levels and ASO titres can be elevated for months and thus the utilisation of a single time point as in this study provides very limited information regarding the streptococcal infection.

Perhaps the most comprehensive case series presented to date was a retrospective study conducted by Demesh et al. (2015) where 10 patients met strict diagnostic criteria for PANDAS [42]. Comparisons were made between parental reports of symptom severity at diagnosis, after antibiotic treatment (*n* = 10) and after tonsillectomy (*n* = 9) using a baseline severity score. Symptom severity improved at all periods after tonsillectomy compared with antibiotics alone. In the surgical cohort, 4 out of nine cases demonstrated a complete resolution, further adding weight to the belief that patients suboptimally treated with antibiotic therapy may benefit from tonsillectomy. However, this study would be subject to recall bias as information was extracted retrospectively from the parents' historical accounts of symptoms using a nonvalidated symptom severity score. Additionally, as the parents had consented on behalf of their child, they would potentially have positively skewed perceptions of the outcome.

In terms of individual cases, Alexander et al. [48] described the case of a 9-year-old boy presenting with ocular facial tics, agitation, and hyperactivity following multiple recurrent GABHS infections. At a routine one-year follow-up following a bilateral tonsillectomy, no further recurrent GABHS infections were reported, and most notably there was a complete resolution of his previous neuropsychiatric symptoms [48]. However, this child was also treated with sertraline prior to tonsillectomy but the report does not clarify whether this medical therapy was stopped after surgery. Hence, outcome may have been the natural progression of the disease in a patient who continued with sertraline for a longer duration of time. Similarly, Batuecas Caletrío et al. [49] described a child with PANDAS (facial tics), following recurrent GABHS, who again, following tonsillectomy, demonstrated a complete resolution of his neuropsychiatric symptoms. Batuecas Caletrío et al. stated that the child had “regular checks” but does not mention the duration of follow-up, thus making it difficult to conclude whether remission occurred instantly or two years later.

Fusco et al. presented the case of an 11-year-old boy, who developed severe choreic movements and satisfied the criteria for the diagnosis of PANDAS [50]. Although the paper reported a complete remission in neurological symptomology following tonsillectomy, cessation in neurological symptomology could not be purely linked to surgical intervention due to the initial initiation of twice-daily tetrabenazine and the long-term use of prophylactic antibiotics. ASO titres levels were reported to be in the normal range following surgical intervention, but, as previously described, the reliability of this finding remains doubtful [37]. 

 Orvidas and Slattery (2001) [51] presented the first case of PANDAS in siblings, with recurrent GABHS pharyngitis despite antibiotic treatment. The presenting complaint was behavioral alterations, mainly tics and OCS, coexistent with recurrent infective bouts. Following tonsillectomy, both siblings exhibited a marked improvement in GAS pharyngitis recurrences and OCS/tic exacerbation at 11-month follow-up. Similarly, Heubi and Shott (2003) reviewed the case of two brothers, one with OCD and the other with a tic disorder, both of whom improved significantly after undergoing adenotonsillectomy for treatment of their recurrent tonsillitis [52]. Doubts regarding the underlying diagnosis, principally due to minimally raised ASO levels in one of the two cases, and an absence of the behavioural oscillations crucially linked to PANDAS therefore question the reliability of the diagnosis.

## 5. Limitations of Literature

There are numerous generic limitations to this sparse literature. First of all, there are incomplete data in several reports regarding the timespan to complete resolution. For example, Bautecus et al. states complete resolution at “regular checks” which provides no relevant information regarding the timespan to complete resolution. Secondly, for the majority of these cases, single measurements of ASO titres and antibody levels were conducted which provide limited information on streptococcal infections. Additionally, the studies utilised various laboratories measures with different upper limits and thus could make drawing conclusions more difficult. The concomitant use of other medical therapy such as antibiotics and neuropsychiatric drugs makes it difficult to state surgery as the sole reason for PANDAS resolution. Furthermore, none of the studies provided information regarding the duration and severity of the neuropsychiatric symptoms prior to intervention.

## 6. Conclusion

PANDAS has become an evolving area of research for a possible relationship, investigating the relationship between GABHS infections in children and the development of either an obsessive-compulsive disorder or tic disorder. Overall, the limited series and case reports in the literature provide an unsubstantiated link between tonsillectomy and symptom remission, predominantly as other notable variables including pharmacological therapy (antibiotics and tetrabenazine), and stress/fatigue were not accounted for. Although deemed as relatively small risk procedure, complications of tonsillectomy must also be considered into the debate advocating surgical intervention. Therefore adenotonsillectomy as a potential therapeutic strategy may only be appropriate in children with antibiotic-refractory PANDAS.

PANDAS is a significantly debilitating condition, with detrimental effects on a child's psychophysical development. This review provides an insight into the current literature and the potential for surgery to treat PANDAS. However due to the current limitations of the literature drawing definitive conclusions is difficult. Thus, the next required steps would involve conducting a prospective collaborative study.

## Figures and Tables

**Table 1:** Summary of case series.

Author	Patient cohort	PANDAS criteria met (including confirmation of GABHS)	Neuropsychiatric symptoms	Data Collation Method	ASOT Threshold	Treatment Provided Prior to Tonsillectomy	Follow-Up Duration after Tonsillectomy	Outcome
Demesh et al. [2015]	Total cohort: *n* = 10 (8 male, 2 female) Average age: 6.5 years Tonsillectomy or adenotonsillectomy: *n* = 9	Yes	Specifics: Tics (*n* = 9) Anxiety/OCD symptoms (*n* = 4)	Retrospective Study 9-item questionnaire (assessed medical history, neuropsychiatric symptoms, symptom response to treatment)	Not reported	All patients were treated with antibiotics in the past or were receiving antibiotic prophylaxis. (Antibiotics used were not reported)	3 months 6 months 1 year 3 years	Reduced symptom severity in all 9 patients who underwent Tonsillectomy Complete resolution in 4 cases

Pavone et al. [2014]	Total cohort: *n* = 120 (57 male, 63 female) Average age: 11.05 ± 1.02 years Tonsillectomy: *n* = 25 Adenotonsillectomy: *n* = 31	Yes		Prospective Study Children's Yale-Brown Obsessive Compulsive Scale (CY-BOCS) Yale Global Tic Severity Scale (YGTSS)	Normal if <266	Antibiotic therapy when inflammatory markers raised or if clinical condition required 3rd-generation cephalosporins (5–7 days BD) or azithromycin (3–5 days OD) IVIG used in 8 patients	Every 2 months for 2 years	No difference in remission rates, progression of syndrome, relapse rates, antibody levels, ASOT levels, anti-DNAse B levels

Murphy et al. [2013]	Total cohort: *n* = 43 (30 male, 13 female) Average age: 9.2 ± 2.4 years Tonsillectomy or adenotonsillectomy: *n* = 36	Yes	OCD/anxiety Tics Motor abnormalities	Prospective Study Children's Yale-Brown Obsessive Compulsive Scale (CY-BOCS) Yale Global Tic Severity Scale (YGTSS)	Elevated if >200	Not reported (Multiple antibiotic courses)	1 year	No difference in remission rates, progression of neuropsychiatric symptoms, ASOT levels, anti-DNAse B and anti-ACHO levels

**Table 2:** Summary of Case Reports.

Author	Patient demographic	PANDAS Criteria Met (including confirmation of GABHS)	Neuropsychiatric symptoms	ASOT titre	Treatment provided prior to tonsillectomy	Follow-Up duration after tonsillectomy	Outcome at follow-up
Alexander et al. [2011]	9-year-old, male	Yes	Oculofacial tics, agitation, hyperactivity	Positive Throat Culture No value reported	Cephalosporin, azithromycin and prophylactic penicillin Sertraline	1 year	Complete resolution

Fusco et al. [2010]	11-year-old, male	Yes	Severe choreic movements of upper limbs Oculo-facial tics	Initial presentation: 694 U/ml 6 months postantibiotics (penicillin + diaminocillina): 782 U/ml 5 months after tetrabenazine: 658 U/ml 8 weeks post-tonsillectomy and 2 weeks after discontinuation of tetrabenazine: 156 U/ml NOTE: Normal range <200 U/ml	Penicillin with monthly diaminocillina Tetrabenazine	3 years	Complete resolution

Batuecas Caletrío et al. [2008]	9-year-old, male	Yes	Oculo-facial tics Obstructive Sleep Apnoea (OSA)	550 *μ*m/ml (elevated)	Not applicable as indication for tonsillectomy was OSA	Not reported	Complete resolution

Lynch et al. [2006]	6-year-old, male	Yes	Facial tics, hemichorea	360	Numerous courses of penicillin	18 months	Complete resolution

Heubi and Shott [2003]	9-year-old, male	Yes	Motor and vocal tics	195 Units NOTE: Normal <160 units	Clonidine	2 months	Almost complete resolution
	10-year-old, male	No	Obsessive-compulsive symptoms	Not reported	Sertraline	1 year	Complete resolution & discharge from psychiatrist

Orvidas and Slattery [2001]	12-year-old, female	Yes	Obsessive-compulsive symptoms	1 : 340 (raised) Positive RADT Test	Azithromycin (intermittent benefit)	11 months	Almost complete resolution (1 acute panic attack)
	8-year-old, male	Yes	Intermittent motor tics, head jerking and ocular tics	Positive RADT Test on three occasions No value reported	Azithromycin (Intermittent benefit)	11 months	Complete resolution

## References

[1] Choby B. A. (2009). Diagnosis and treatment of streptococcal pharyngitis. *Fam Physician*.

[2] Barzilai A., Miron D., Sela S. (2001). Etiology and management of acute and recurrent group a Streptococcal tonsillitis. *Current Infectious Disease Reports*.

[3] Efstratiou A., Lamagni T. (2016). *Epidemiology of Streptococcus pyogenes*.

[4] Shaikh N., Leonard E., Martin J. M. (2010). Prevalence of streptococcal pharyngitis and streptococcal carriage in children: A meta-analysis. *Pediatrics*.

[5] Danchin M. H., Rogers S., Selvaraj G. (2004). The burden of group A streptococcal pharyngitis in Melbourne families. *Indian J Med Res*.

[6] Kunnamo A., Korppi M., Helminen M. (2016). Tonsillitis in children: unnecessary laboratory studies and antibiotic use. *World Journal of Pediatrics*.

[7] Lin S., Kaplan E. L., Rao X. (2008). A school-based program for control of group A streptococcal upper respiratory tract infections: A controlled trial in Southern China. *The Pediatric Infectious Disease Journal*.

[8] Martin J. M., Green M., Barbadora K. A., Wald E. R. (2004). Group a streptococci among school-aged children: Clinical characteristics and the carrier state. *Pediatrics*.

[9] Principi N., Marchisio P., Calanchi A. (1990). Drugs ExpClin Res. *Drugs ExpClin Res*.

[10] Swedo S. E., Leonard H. L., Garvey M. (1998). Paediatric autoimmune neuropsychiatric disorders associated with streptococcal infections: clinical description of the first 50 cases. *Am J Psychiatry*.

[11] Kurlan R., Kaplan E. L. (2004). The pediatric autoimmune disorders associated with streptococcal infection (PANDAS) etiology for tics and obsessive-compulsive symptoms: hypothesis or entity? practical considerations for the clinician. *Pediatrics*.

[12] Leonard H. L, Swedo S. E. (2001). Paediatric autoimmune neuropsychiatric disorders associated with streptococcal infection (PANDAS). *International Journal of Neuropsychopharmacy*.

[13] Pichichero M. E. (2009). The PANDAS syndrome. *Hot Topics in Infection and Immunity in Children*.

[14] Giuliano J. D., Zimmerman A., Walkup J. T., Singer H. S. (1998). Prevalence of pediatric autoimmune neuropsychiatric disorders associated with streptococcal infection by history in a consecutive series of community referred children evaluated for tics. *Ann Neurol*.

[15] Cardona F., Orefici G. (2001). Group A streptococcal infections and tic disorders in an Italian pediatric population. *Journal of Pediatrics*.

[16] Olsen R. J., Shelburne S. A., Musser J. M. (2009). Molecular mechanisms underlying group A streptococcal pathogenesis. *Cellular Microbiology*.

[17] Bisno A. L., Gerber M. A., Gwaltney J. M., Kaplan E. L., Schwartz R. H. (2002). Practice guidelines for the diagnosis and management of group A streptococcal pharyngitis. *Clinical Infectious Diseases*.

[18] Courtney H. S., Hasty D. L., Dale J. B. (2002). Molecular mechanisms of adhesion, colonization, and invasion of group A streptococci. *Annals of Medicine*.

[19] Kang H. J., Coulibaly F., Clow F., Proft T., Baker E. N. (2007). Stabilizing isopeptide bonds revealed in gram-positive bacterial pilus structure. *Science*.

[20] Gough H., Luke G. A., Beeley J. A., Geddes D. A. M. (1996). Human salivary glucose analysis by high-performance ion-exchange chromatography and pulsed amperometric detection. *Archives of Oral Biolog*.

[21] Shelburne S. A., Keith D. B., Davenport M. T., Horstmann N., Brennan R. G., Musser J. M. (2008). Molecular characterization of group A Streptococcus maltodextrin catabolism and its role in pharyngitis. *Molecular Microbiology*.

[22] Virtaneva K., Porcella S. F., Graham M. R. (2005). Longitudinal analysis of the group A Streptococcus transcriptome in experimental pharyngitis in cynomolgus macaques. *Proceedings of the National Acadamy of Sciences of the United States of America*.

[23] Hoe N. P., Ireland R. M., DeLeo F. R. (2002). Insight into the molecular basis of pathogen abundance: Group A Streptococcus inhibitor of complement inhibits bacterial adherence and internalization into human cells. *Proceedings of the National Acadamy of Sciences of the United States of America*.

[24] Lim M., Vincent A. (2012). More movements in neuroimmunology. *Brain*.

[25] Jain V. (2010). Inflammatory and autoimmune disorders of the nervous system in children. *Clinics in Developmental Medicine*.

[26] Kirvan C. A., Swedo S. E., Snider L. A., Cunningham M. W. (2006). Antibody-mediated neuronal cell signaling in behavior and movement disorders. *Journal of Neuroimmunology*.

[27] Dale R. C., Church A. J., Surtees R. A. H. (2004). Encephalitis lethargica syndrome: 20 new cases and evidence of basal ganglia autoimmunity. *Brain*.

[28] Vincent A. (2004). Encephalitis lethargica: Part of a spectrum of post-streptococcal autoimmune diseases?. *Brain*.

[29] Dale R. C., Candler P. M., Church A. J., Wait R., Pocock J. M., Giovannoni G. (2006). Neuronal surface glycolytic enzymes are autoantigen targets in post-streptococcal autoimmune CNS disease. *Journal of Neuroimmunology*.

[30] Brimberg L., Benhar I., Mascaro-Blanco A. (2012). Behavioral, pharmacological, and immunological abnormalities after streptococcal exposure: A novel rat model of sydenham chorea and related neuropsychiatric disorders. *Neuropsychopharmacology*.

[31] Macerollo A., Martino D. (2013). *Pediatric Autoimmune Neuropsychiatric Disorders Associated with Streptococcal Infections (PANDAS): An Evolving Concept*.

[32] Dale R. C., Merheb V., Pillai S. (2012). Antibodies to surface dopamine-2 receptor in autoimmune movement and psychiatric disorders. *Brain*.

[33] Felsenstein S., Faddoul D., Sposto R., Batoon K., Polanco C. M., Bard J. D. (2014). Molecular and clinical diagnosis of group A streptococcal pharyngitis in children. *Journal of Clinical Microbiology*.

[34] Lean W. L., Arnup S., Danchin M., Steer A. C. (2014). Rapid diagnostic tests for group a streptococcal pharyngitis: A meta-analysis. *Pediatrics*.

[35] Cohen J. F., Bertille N., Cohen R., Chalumeau M. (2016). Rapid antigen detection test for group A streptococcus in children with pharyngitis. *Cochrane Database of Systematic Reviews*.

[36] Choby B. A. (2009). Diagnosis and treatment of streptococcal pharyngitis. *American Family Physician*.

[37] Blyth C. C., Robertson P. W. (2006). Anti-streptococcal antibodies in the diagnosis of acute and post-streptococcal disease: streptokinase versus streptolysin O and deoxyribonuclease B. *Pathology*.

[38] Singer H. S., Gilbert D. L., Wolf D. S., Mink J. W., Kurlan R. (2012). Moving from PANDAS to CANS. *Journal of Pediatrics*.

[39] E. Swedo S. (2012). From research subgroup to clinical syndrome: modifying the PANDAS criteria to describe pans (pediatric acute-onset neuropsychiatric syndrome). *Pediatrics & Therapeutics*.

[40] Shaikh N., Leonard E., Martin J. M. (2010). Prevalence of streptococcal pharyngitis and streptococcal carriage in children: A meta-analysis. *Pediatrics*.

[41] Swedo S. E. (1994). Sydenham's Chorea: A Model for Childhood Autoimmune Neuropsychiatric Disorders. *Journal of the American Medical Association*.

[42] Demesh D., Virbalas J. M., Bent J. P. (2015). The role of tonsillectomy in the treatment of Pediatric Autoimmune Neuropsychiatric Disorders Associated with Streptococcal Infections (PANDAS). *JAMA Otolaryngology - Head and Neck Surgery*.

[43] Blakley B. W., Magit A. E. (2009). The role of tonsillectomy in reducing recurrent pharyngitis: A systematic review. *Otolaryngology—Head and Neck Surgery*.

[44] Orvidas L. J., St. Sauver J. L., Weaver A. L. (2006). Efficacy of tonsillectomy in treatment of recurrent group A *β*-hemolytic streptococcal pharyngitis. *The Laryngoscope*.

[45] Murphy T. K., Lewin A. B., Parker-Athill E. C., Storch E. A., Mutch P. J. (2013). Tonsillectomies and adenoidectomies do not prevent the onset of pediatric autoimmune neuropsychiatric disorder associated with group a streptococcus. *The Pediatric Infectious Disease Journal*.

[46] Kaplan E. L., Rothermel C. D., Johnson D. R. (1998). Antistreptolysin O and anti-deoxyribonuclease B titers: normal values for children ages 2 to 12 in the United States. *Pediatrics*.

[47] Pavone P., Rapisarda V., Serra A. (2014). Pediatric autoimmune neuropsychiatry disorder associated with group a streptococcal infection: The role of surgical treatment. *International Journal of Immunopathology and Pharmacology*.

[48] Alexander A. A. Z., Patel N. J., Southammakosane C. A., Mortensen M. M. (2011). Pediatric autoimmune neuropsychiatric disorders associated with streptococcal infections (PANDAS): An indication for tonsillectomy. *International Journal of Pediatric Otorhinolaryngology*.

[49] Batuecas Caletrío Á., Sánchez González F., Cruz Ruiz S. S., Santos Gorjón P., Blanco Pérez P. (2008). PANDAS Syndrome: ANew Tonsillectomy Indication?. *Acta Otorrinolaringologica (English Edition)*.

[50] Fusco F. R., Pompa A., Bernardi G. (2010). A case of PANDAS treated with tetrabenazine and tonsillectomy. *Journal of Child Neurology*.

[51] Orvidas L. J., Slattery M. J. (2001). Pediatric autoimmune neuropsychiatric disorders and streptococcal infections: Role of otolaryngologist. *The Laryngoscope*.

[52] Heubi C., Shott S. R. (2003). PANDAS: Pediatric autoimmune neuropsychiatric disorders associated with streptococcal infections - An uncommon, but important indication for tonsillectomy. *International Journal of Pediatric Otorhinolaryngology*.

